# Relapsing facial pemphigus vegetans associated with chronic intranasal cocaine use

**DOI:** 10.1016/j.jdcr.2026.05.038

**Published:** 2026-05-22

**Authors:** Wisoo Shin, Richard M. Haber, Charlene Hunter, Habib A. Kurwa, Sabrina Nurmohamed, Lynne H. Robertson

**Affiliations:** a Division of Dermatology, Department of Medicine, University of Calgary, Calgary, Alberta, Canada; b Department of Pathology and Laboratory Medicine, University of Calgary, Calgary, Alberta, Canada; c Department of Dermatology and Skin Science, University of British Columbia, Vancouver, British Columbia, Canada

**Keywords:** cocaine, drug-induced pemphigus pemphigus, pemphigus vegetans

## Background

Pemphigus is a group of autoimmune mucocutaneous vesiculobullous diseases that are potentially fatal.^1^ Pemphigus vegetans is a rare subtype of pemphigus vulgaris characterized by flaccid blisters which evolve into fungoid vegetative plaques involving the intertriginous areas, scalp, and oral mucosa.^1^ Thiol and phenol drugs such as D-penicillamine and aspirin have been implicated in drug-induced pemphigus.^2^^,^^3^ A growing number of reports have linked intranasal consumption of cocaine and pemphigus.^3^, ^4^, ^5^, ^6^, ^7^ We reported a case of cocaine-associated centrofacial pemphigus vegetans involving the entire lower face due to chronic intranasal cocaine use, further strengthening the relationship between cocaine and drug-induced pemphigus.

## Case report

A 45-year-old man with a longstanding history of intranasal cocaine use and alcohol use disorder was hospitalized for an undiagnosed eroded and crusted edematous plaque involving the distal third of the nose and upper cutaneous lip persisting over 5 months. The lesion appeared approximately 1 month after exposure to intranasal cocaine through a lacerated nasal cavity. No other illicit drug or medication exposures occurred prior to the onset of the facial lesion.

Prior to admission, he was unsuccessfully treated with several courses of systemic antibiotics (cephalexin, cefazolin and probenecid, amoxicillin-clavulanic acid, clindamycin, and trimethoprim/sulfamethoxazole) for a working diagnosis of chronic soft tissue infection. Methicillin-sensitive *Staphylococcus aureus* was the only organism grown from bacterial swabs and bacterial tissue cultures, while fungal and mycobacterial cultures were negative. Three sets of biopsies by the outpatient Ear, Nose, and Throat team from the nasal plaque taken during the 5-month period showed nonspecific mixed inflammatory infiltrates with no evidence of a neoplastic/malignant or infectious process.

In the hospital, laboratory tests were significant for anemia (129 g/L) and mild hypoalbuminemia (29 g/L), with normal levels of creatinine, electrolytes, C-reactive protein, bilirubin, and liver enzymes. Serologic testing for syphilis, human immunodeficiency virus, and hepatitis B and C virus was negative. Epstein-Barr virus polymerase chain reaction and IgG were positive, suggestive of an incidental concurrent chronic infection. An autoimmune panel revealed an antinuclear antibody titer of 1:80 (centrosome pattern). Extractable nuclear antigen, anti-double-stranded DNA, and antineutrophil cytoplasmic antibodies were negative with normal complement levels. A computed tomography (CT) scan of facial bones revealed a large homogenous mass-like soft tissue thickening of the nasal skin without signs of bone destruction or abscesses. Additional CT scans of the chest/abdomen/pelvis were unremarkable.

**Fig 1:**
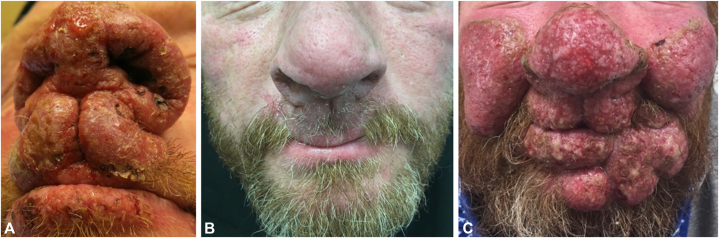
**A**, Clinical presentation of a 45-year-old man with a vegetative eroded and crusted plaque involving the distal nose and upper cutaneous lip. **B,** Near clearance of the lesion within 1 month of treatment with oral prednisone. **C,** Acute flare and worsening of the lesion in 2.5 months with inconsistent prednisone use.

**Fig 2:**
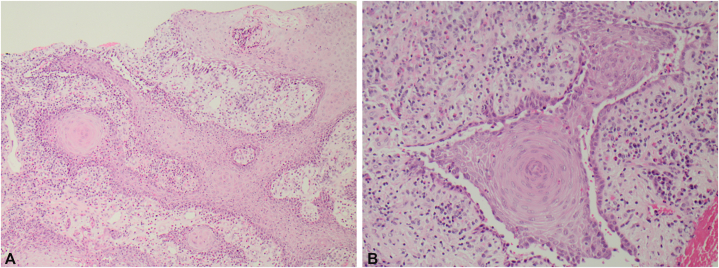
**A**, Low power image shows marked irregular epidermal acanthosis with suprabasilar acantholysis and dense associated eosinophil-rich infiltrate. **B,** High power image shows pseudoepitheliomatous epithelial hyperplasia with suprabasilar acantholysis and an eosinophil-rich infiltrate. (**A** and **B,** Hematoxylin-eosin stain; original magnifications: **A,** ×10; **B,** ×20.)

On dermatology assessment, a thick vegetative confluent plaque with numerous erosions and crusts involving the nasal tip, bilateral nares, columella, and upper cutaneous lip was observed (Fig 1, *A*). Repeat biopsies of the nasal plaque showed suprabasal acantholysis involving the follicular epithelium (Fig 2), and direct immunofluorescence (DIF) on tissue collected in normal saline was positive for patchy intercellular C3 within the follicular epithelium. IgA, IgG, and immunoglobulin M were negative. The patient declined multiple requests for further testing, including indirect immunofluorescence for desmoglein 1 and 3 antibodies. Warthin-Starry, Gram, Grocott, Ziehl-Neelsen, and periodic acid–Schiff stains were negative.

A diagnosis of pemphigus vegetans was made based on histological findings and clinical features. He was subsequently started on prednisone 80 mg orally daily, and the lesions entirely cleared within 1 month of treatment compliance (Fig 1, *B*). However, the uninsured patient could not afford regular doses of oral prednisone and was unable to discontinue cocaine consumption despite ongoing counseling. As a result, his disease flared within 2.5 months and progressed into pustular and exudative crusted plaques involving the entire lower face (Fig 1, *C*) with inconsistent use of clinic-provided oral prednisone and irregular intralesional triamcinolone (10 mg/mL) injections. The patient was lost to dermatology follow-up after 2 years of fluctuating disease and inconsistent follow-up. He passed away from a cardiac arrest, 5 years after initial contact.

## Discussion

To our best knowledge, this is the second case of cocaine-associated pemphigus vegetans.^3^ Table I^3^, ^4^, ^5^, ^6^, ^7^, ^8^, ^9^ summarizes English reported cases of cocaine-associated pemphigus.^4^, ^5^, ^6^, ^7^ The striking evolution of disease involving the inferior half of the face and oral mucosa (Fig 1, *C*) has not been reported.

**Table I:** Summary of case reports and series indicating association of pemphigus and cocaine consumption

Cases	Diagnosis	Patient age/sex	Areas of involvement	Pathology, hematoxylin-eosin staining	Pathology DIF	Desmoglein antibodies	Effective therapy	Maintenance therapy	Resolution	Complications
Laguna et al^4^ 2008	Cocaine- associated pemphigus vulgaris	37 yr/M	Back, oral and genital mucosa	Intraepidermal vesicles with suprabasal acantholysis	Intercellular IgG and C3	Anti-ICS 1/40	Prednisone 90 mg orally daily and MMF 1 g orally daily	Prednisone 60 mg orally daily and MMF 1 g daily	Improvement after cocaine cessation	Relapse × 2
Ngo et al^3^ 2012	Cocaine- associated pemphigus vegetans	42 y/M	External nares, upper lips, inguinal folds, thumb	Pseudoepitheliomatous epidermal hyperplasia with epidermal pustules containing eosinophils and neutrophils, and extensive suprabasal acantholysis	Intercellular C3 deposits	Negative	Prednisone 80 mg orally daily and MMF 1.5 mg orally twice a day	Nil	Complete resolution over 18 m	Nil
Jiménez-Zarazúa et al^5^ 2018	Cocaine-associated pemphigus vulgaris	33 y/M	BSA 35%, oral cavity, thorax, inguinal and genital regions	Suprabasal bullous lesions and acantholysis	Not reported	Unable to obtain due to lack of availability	Methylprednisolone 500 mg IV and cyclophosphamide 500 mg IV every 15 d, prednisone 50 mg orally daily, mycophenolic acid 500 mg orally 4 times a day	Prednisone 25 mg orally daily	Complete resolution in 86 wk	Relapse × 10
Arisi et al^6^ 2019	Cocaine-associated pemphigus vulgaris	43 y/M	Oral mucosa and lower lip	Intraepidermal vesicles with suprabasal acantholysis	Intercellular C3 deposits	Anti-Dsg3 positive (30 U/mL)	IVIG 400 mg/kg/d, G-CSF and ceftriaxone	Prednisone orally	Minor improvement in 2 mo	Hospitalization, Relapse × 1
Juhasz et a^7^ 2021	Cocaine-associated pemphigus vulgaris	31 y/M	BSA 70%, head, oral mucosa, neck, trunk and all extremities	Suprabasal acantholysis	Epithelial surface IgG deposits	IgG anti-Dsg1 6100 U and IgG anti-Dsg3 4600 U	IVIG 2 g/kg over 4 days, prednisone 70 mg orally daily, MMF 1 g twice a day, niacinamide 500 mg orally 3 times a day, doxycycline 100 mg orally twice a day, topical clobetasol 0.05%	Same as effective therapy, with IVIG monthly	Significant improvement in 6 wk	Hospitalization, *Pseudomonas aeruginos*a infection
Fabregat-Pratdepadua et al^8^ 2022 Case 1	Cocaine-associated PSV-PDV	37 y/F	Nasal vestibule, philtrum and lips, intertriginous areas and genitals	Epidermal hyperplasia, eosinophilic and neutrophilic intraepidermal microabscesses	IgA and IgG at the basement membrane	Negative	Doxycycline and rifampicin, corticosteroids 0.5 mg/kg/d	Nil	Improvement in 2 mo	Nil
Fabregat-Pratdepadua et al^8^ 2022 Case 2	Cocaine-associated PSV-PDV	53 y/M	Root of the nose	Similar to case 1	Negative	Negative	Doxycycline and rifampicin, corticosteroids 0.5 mg/kg/d	Nil	Improvement in 2 mo	Nil
Pattison et al^9^ 2024	CIMDL	55 y/F	Right nare and cutaneous lip	Lymphoplasmacytic proliferation (CD138+ plasma cells and polytypic k and λ immunoglobulin light chains. No eosinophilic microabscess)	Not performed	Negative	Not reported	Nil	Not reported	Nil
This case	Cocaine-associated pemphigus vegetans	45 y/M	Entire lower half of the face	Suprabasal acantholysis	Intercellular C3 deposits	Not completed	Prednisone 80 mg orally daily with intralesional triamcinolone (10 mg/L)	Prednisone 50 mg orally daily	Significant improvement within 1 mo, lost to follow-up	Relapse × 1

*BSA,* Body surface area; *CD*, Cluster of differentiation; *CIMDL,* cocaine-induced midline destructive lesion; *DIF,* direct immunofluorescence; *F*, female; *G-CSF*, granulocyte colony stimulating factor; *ICS,* intercellular substance; *M*, male; *MMF,* mycophenolate mofetil; *IV,* intravenously; *IVIG,* intravenous immunoglobulin; *PDV*, pyodermatitis vegetans; *PSV*, pyostomatitis vegetans.

The mechanism of cocaine-induced pemphigus remains speculative. Pathogenesis is thought to be linked to the compound's complex chemical nature, including concomitant adulterants and degradation products.^3^^,^^4^ It is hypothesized the hydrolysis of cocaine may generate phenol-containing intermediates, driving acantholysis.^2^^,^^3^ Furthermore, coadministered contaminants found in intranasal preparations such as levamisole, quinine, procaine, and amphetamines may also contribute to disease.^4^^,^^7^ These substances, alongside processing irritants like talc, borax, or hydrochloric acid, may act as potent chemical or physical triggers.^3^ Given that our group reported the only other case of cocaine-associated pemphigus vegetans, a locally distributed diluent or additive should be considered as a potential precipitant.

Localized vegetative lesions involving the nose and upper lip present a diagnostic challenge with a differential diagnosis of pemphigus (vegetans and erythematosus), herpes simplex infection, bacterial infections including rhinoscleroma and blastomycosis-like pyoderma, deep fungal infections, mycobacterial and atypical mycobacterial infections, and lupus erythematosus. Recently, 3 clinical differential diagnoses of cocaine-associated centrofacial vegetative lesions have been described: pyodermatitis-pyostomatitis vegetans (PPV),^8^ cocaine-induced midline destructive lesion,^9^ and cocaine-induced dermatomucositis (CID).^10^ PPV is a rare inflammatory disorder characterized by vegetative exudative vesiculopustular plaques of unknown etiology, which is commonly associated with inflammatory conditions such as inflammatory bowel disease.^8^ CID has been described since 2023, with the development of skin thickening involving the upper lip and nostrils with cocaine use and histologic findings of polyclonal plasma cell infiltrates and IgG4 positivity on DIF.^10^ Thick vegetative and pustular plaques involving the bilateral cheeks and chin developed in our patient. DIF was negative for IgA, IgG or immunoglobulin M, and the CT scan of the chest/abdomen/pelvis and symptoms were negative for signs of inflammatory bowel disease. The overall clinical picture and pathology results support a diagnosis of pemphigus vegetans rather than PPV or CID. There was no evidence of destruction of nasal architecture on the CT scan of the facial bones to suggest a cocaine-induced midline destructive lesion. The lack of dsg1/3 antibodies remains a limitation. However, this resulted from unavoidable constraints given the patient’s deferral.

An increasing number of reports associating pemphigus with intranasal use of illicit drugs such as cocaine and heroin highlights the importance of a complete medication history in clinical evaluation. Achieving long-term remission of cocaine-associated pemphigus requires an integrated approach, combining pharmacologic management (corticosteroids and another immunosuppressant) with multidisciplinary social support for sustainable drug cessation.

## Conflicts of interest

None disclosed.
